# Resistance Profiling of Predominant Non–*E. coli* Enterobacteriaceae Isolated From Humans, Food Animals, and the Environment in the Fako Division of Cameroon

**DOI:** 10.1155/bmri/3947539

**Published:** 2025-06-23

**Authors:** Clovis Elah Ndialle, Mildred Mbom Nyincho, Matil Eyong, Derick Lekealem Nkwetta, Manuel Ritter, Patrick A. Njukeng, Samuel Wanji, Jane-Francis T. K. Akoachere

**Affiliations:** ^1^Department of Microbiology and Parasitology, University of Buea, Buea, Cameroon; ^2^Research Foundation in Tropical Diseases and the Environment, Buea, Cameroon; ^3^Institute for Medical Microbiology, Immunology and Parasitology (IMMIP), University Hospital Bonn (UKB), Bonn, Germany; ^4^German Center for Infection Research (DZIF), Partner Site Bonn Cologne, Bonn, Germany; ^5^GermanWest African Centre for Global Health and Pandemic Prevention (GWAC), Partner Site Bonn, Bonn, Germany

**Keywords:** drug resistance, *Enterobacteriaceae*, environment, Fako Division, food animals, humans

## Abstract

The impact of the current global rising resistance of Enterobacteriaceae to antibiotic agents is of great concern. Detecting and monitoring resistance in these pathogens in humans, animals, and the environment and taking appropriate actions based on results obtained are indispensable to reverse this trend. This study is aimed at contributing to the fight against resistance of predominant non–*Escherichia coli* Enterobacteriaceae in the Fako Division of Cameroon through a one-health approach. Freshly collected human feces, rectal swabs from pigs, cloacal swabs from chicken, cow intestinal content, and environmental samples were cultured. Isolates were identified using API 20E. Predominant non–*E. coli* isolates (*Enterobacter* spp., 65.0%; *Salmonella* spp., 11%; and *Citrobacter* spp., 9.9%) were confirmed by polymerase chain reaction (PCR). Antibiotic susceptibility profiles of these isolates were determined by the Kirby-Bauer disc diffusion technique, while the resistant genes were detected by PCR. The quinolones (norfloxacin, 94.7%, and ofloxacin, 91.2%), carbapenem (imipenem, 96%), aminoglycoside (amikacin, 95.5%), and chloramphenicol (91.3%) were the most active drugs. Penicillins (amoxicillin, 24.7%; ampicillin, 21.2%; and amoxicillin–clavulanic acid, 19.9%) were the most inactive drugs. However, isolates showed the highest rate of intermediate susceptibility (48.6%) to cefepime. Out of the 226 isolates, 214 (94.7%) showed resistance to at least one antibiotic agent. Multidrug resistance was found in 54 (25.2%) of the isolates. The predominant antibiotypes were AX^R^ AM^R^ AMC^R^ (25, 11.1%), AX^R^ AM^R^ AMC^R^ AZM^R^ (18, 8.4%), CAZ^R^ AX^R^ AM^R^ AMC^R^ (12, 5.3%), and AX^R^ AMC^R^ AZM^R^ (7, 3.1%). Isolates with these antibiotypes were from various sources and predominant genera. Plasmid-mediated quinolone resistance (PMQR) genes (*acrA*, *acrB*, *qepA*, and *aac(6*⁣′* )-ib-cr*) were detected in 97.8% (44/45) of isolates that showed resistance to at least one quinolone antibiotic, while the beta-lactamase genes, *blaCMY-2* and *blaCTX-M-1*, were detected in 7.9% (5/63) and 22.0% (14/63), respectively, in isolates that showed resistance to cephalosporins. These isolates carrying these genes were from humans, food animals, and the environment. Of the 45 isolates, a total of 40 (88.9%) carried two or more PMQR genes, while 2 (0.6%) carried both *bla* genes (cocarriage). Five (17.9%) isolates out of the 28 screened for PMQR and beta-lactamase genes were positive for both sets of genes. Resistance to antibiotics was high with strains of the different genera carrying PMQR and beta-lactamase resistance genes circulating in humans, food animals, and the environment in the Fako Division of Cameroon.

## 1. Introduction

The family Enterobacteriaceae are gram-negative, nonspore-forming bacilli. They are part of the normal flora of the intestines of humans and animals and can also be found in the environment (soil, water, plants, and decaying matter) [1]. These bacteria are zoonotic [2] and constitute an important group of human and animal pathogens. They possess a high capacity for developing drug resistance owing to a matrix of plasmid-borne resistance genes they often carry and undergo mutations in the presence of a stressor such as antibiotics [3]. Most antibiotics used as growth enhancers and therapeutic or prophylactic agents in the livestock sector are equally used in human medicine [4], increasing their chances of being misused.

Misuse of antibiotics in the animal industry leads to indirect consumption by humans through their residues in meat products (especially in developing countries where meat consumption is still very high) and contaminated groundwater due to poor waste disposal from animal farms [4, 5]. These residues may produce several consequences, including developing antibiotic-resistant bacteria [6]. The high frequency of multidrug resistance in this family of bacteria especially to the high priority critically important antibiotics coupled with the insufficient discovery of new effective drugs makes these organisms a serious threat to public health [7]. In 2017, blood stream infections caused by carbapenem-resistant Enterobacteriaceae (CRE) accounted for a 25.7% mortality rate in China [8], and the World Health Organisation rates Enterobacteriaceae as the fifth cause of mortality after malaria, HIV-AIDS, neonatal diseases, and lower respiratory diseases in Cameroon [9]. Therefore, understanding the antibiotic resistance patterns of these organisms is important for the clinical management of infections they cause.

In Cameroon, studies have investigated Enterobacteriaceae from food animals [10–12], clinical samples [13–15], and environmental samples [16–18]. Bissong et al. [19] compared the susceptibility of *Escherichia coli* from clinical and zoonotic sources and reported higher resistance rates in clinical than in zoonotic samples. A systematic review and meta-analysis by Mouiche et al. [11] presented data on the resistance profile of Enterobacteriaceae and non-Enterobacteriaceae from some studies carried out in different regions of Cameroon. None of these studies investigated these organisms following the one-health concept. There is more data on *E. coli* than on other members of the Enterobacteriaceae in our study area, which is the reason why our study focused on non–*E. coli* Enterobacteriaceae. The scarcity of research data on antibiotic resistance profiles for species of non–*E. coli* Enterobacteriaceae makes empirical treatment challenging as most clinical settings in our study area lack laboratory facilities for antimicrobial susceptibility testing. Establishing the occurrence and antibiotic resistance profiles of these potential pathogens in humans, animals, and the environment is essential for establishing effective treatment, prevention, and control measures. In the face of this dilemma, our study is aimed at producing data on the susceptibility pattern and antibiotic resistance genes of non–*E*. *coli* Enterobacteriaceae in isolates from humans, food animals, and the environment. Such information will help measure the degree of resistance stemming from these pathogens and identify effective antibiotics for clinical and veterinary care.

## 2. Materials and Methods

### 2.1. Study Design

This was a cross-sectional study in which the participating health facilities, abattoirs, and animal farms were selected by convenience sampling. Sample collection spanned from March to September 2020. Non–*E. coli* Enterobacteriaceae were isolated, and antibiotic resistance profiles as well as resistance genes of predominant isolates were investigated.

### 2.2. Study Site

**Figure 1:**
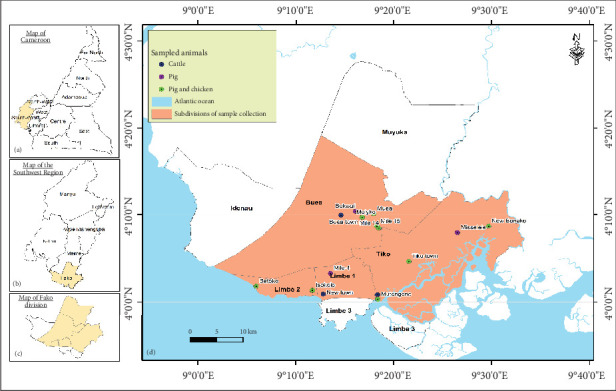
Map of sample collection sites.

This study was conducted in Buea, Limbe, Mutengene, and Tiko, which are major towns of the Fako Division, Cameroon. Administratively, Fako is one of the six divisions of the Southwest Region of Cameroon. The headquarters are in Limbe. Fako Division also harbours Buea, the regional headquarters of the Southwest Region.  Figure 1 shows the location of Fako Division and the sites where samples were collected. With regard to its health structure, Fako Division has four health districts, namely, Limbe, Tiko, Buea, and Muyuka.

### 2.3. Sample Collection

Freshly produced stool samples were collected from humans at the Limbe Regional Hospital, Mutengene Sub-Divisional Medical Centre, Tiko CDC Central Clinic, and the Buea Regional Hospital. A sterile swab was inserted 2–3 cm into the rectum of pigs, cloacae of chicken, and intestine of cattle during slaughter, rotated three to five times clockwise and withdrawn. Surfaces in the hospital environment (sink, bedside cupboard, ward table, doorknobs, bed rail, and floor) were swabbed. Water samples were collected at different points in streams flowing in Buea, Mutengene, Tiko, and Limbe using a sterile syringe. Samples were also collected from knives, cutlasses, soil, and the floor at slaughterhouses and from drinkers, feeders, drinking water, soil, and the floor of pig and poultry farms by swabbing their surfaces. The sample collection respected aseptic techniques.

One–two grams of human stool, swabs carrying samples, and 2 mL of water sample were placed in 10 mL of selenite F broth and in nutrient broth and transported to the laboratory following standard procedures [20].

### 2.4. Bacteriological Analysis

Samples were incubated for 18–24 h at 37°C, after which a loop full of each broth culture was aseptically streaked on cysteine lactose electrolyte deficient (CLED) agar. Plates were incubated for 18–24 h at 37°C for isolation of Enterobacteriaceae. All large, elevated, yellow, opaque colonies with a centre more intense yellow on yellowish medium were considered *E. coli* [21] and eliminated from the study. Every other colony was streaked on nutrient agar plates to obtain pure cultures which were subjected to oxidase testing, catalase testing, and gram staining. The gram-negative, oxidase-negative, and catalase-positive bacilli were then identified using analytical profile index (API) 20E (bioMérieux, Marcy-l'Étoile, France) following the manufacturers' instructions. Predominant isolates were confirmed with PCR, using Bio-Rad T100 TM Thermal Cycler.

#### 2.4.1. Confirmation of the Identity of Isolates

To confirm the identity of isolates obtained with API 20E, PCR was done for 30 randomly selected *Enterobacter* isolates and for 10 each of *Salmonella* and *Citrobacter* isolates. Bacterial DNA was extracted using the SDS chloroform protocol described by Sambrook and Russell [22]. Amplification was achieved by conventional PCR in a 20 *μ*L final reaction mixture. Each reaction tube contained 10 *μ*L master mix, 8 *μ*L nuclease-free water, 0.5 *μ*L of each primer (forward and reverse), and 1 *μ*L DNA template. Amplification conditions are presented in Table 1. Amplicons were loaded on agarose gel alongside a 1-kb Plus Ladder (Thermo Fisher Scientific), and electrophoresis was performed at 120 V and 400 mA for 45 min. Detection was done with the Gel Doc XR+ imaging system (Bio-Rad).

### 2.5. Antibiotic Susceptibility Testing (AST)

Predominant Enterobacteriaceae isolates were subjected to AST by the Kirby-Bauer disc diffusion technique as described by Bauer et al. and CLSI [26, 27]. Two to three colonies of pure isolates were emulsified in sterile normal saline in a bijou bottle and adjusted to a density of 0.5 McFarland standard which is approximately equal to 1.5 × 10^8^ bacteria/mL. A uniform lawn of standard suspension of bacterial isolates was made on Mueller–Hinton agar. The following antibiotic discs (Bioanalyse, Ve Tic. Ltd., Sti, Yenimahalle Turkey) were tested: amoxicillin–clavulanic acid (AMC), 30 *μ*g; amoxicillin (AX), 30 *μ*g; ampicillin (AM), 30 *μ*g; imipenem (IPM), 10 *μ*g; cefixime (CFM), 5 *μ*g; ceftazidime (CAZ), 30 *μ*g; cefotaxime (CTX), 30 *μ*g; ceftriaxone (CRO), 5 *μ*g; cefepime (FEP), 10 *μ*g; aztreonam (ATM), 30 *μ*g; chloramphenicol C, 30 *μ*g; azithromycin (AZM), 15 *μ*g; tetracyclines (TEs), 30 *μ*g; cotrimoxazole (SXT), 25 *μ*g; norfloxacin (NOR), 10 *μ*g; nalidixic acid (NA), 30 *μ*g; ciprofloxacin (CIP), 5 *μ*g; ofloxacin (OFX), 5 *μ*g; and amikacin (AK), 30 *μ*g. The antibiotics used were selected based on local practice and literature [28, 29]. Discs were placed at least 25 mm apart and 10 mm from the edge of the plate, to avoid overlapping of the zones of inhibition.

Plates were incubated for 18–24 h at 37°C, and the diameters of the zones of inhibition were measured and recorded. Isolates were either classified as sensitive, intermediate, or resistant to each of the antibiotics based on the interpretive category for zone diameter breakpoints achieved using the CLSI M100-Ed 31 [27] and the British Society for Antimicrobial Chemotherapy [28] (for AX).

### 2.6. Screening for Resistance Genes

Isolates resistant to quinolones and cephalosporins were screened for plasmid-mediated quinolone resistance (PMQR) and the beta-lactamase resistance (bla) genes, by conventional PCR using specific primers (Table 2). The PMQR genes screened were the *qepA*, *acrA*, *acrB* and *aac(6*⁣′* )-ib-cr*, while the beta-lactamase genes screened were the *blaCMY-2* and *blaCTX-M-1*. Bacterial plasmid was extracted with the miniprep protocol as described by Birnboim and Doly [29], and the amplification was done in a 20 *μ*L final reaction volume using the amplification conditions shown on Table 2. Amplicons were detected as described under bacteriological analysis.

### 2.7. Statistical Analysis

Data was expressed as percentages. The analysis of variance (ANOVA) was used to compare differences in the distribution of isolates among the various sources. The level of significance was set at *p* ≤ 0.05.

## 3. Results

### 3.1. Summary of Samples Collected

A total of 2042 samples were collected as follows: 976 (47.8%) from humans, 743 (36.4%) from animals, and 323 (15.8%) from the environment (Table 3). The distribution of environmental samples is presented in Table 4.

### 3.2. Prevalence of Non–*E. coli* Enterobacteriaceae

Out of the 2042 samples collected, a total of 260 non–*E. coli* isolates belonging to 16 genera (Table 5) were identified using API 20E. Species identification was achieved for 156 (60.0%) of the isolates. Most (97, 37.3%) of the isolates were from humans, while the least were from pigs (24, 9.2%). There was no significant difference in the distribution of the isolates among the different sources (*p* = 0.7).

The predominant genera were *Enterobacter* (171, 65.8%), *Salmonella* (29, 11.2%), and *Citrobacter* (26, 10%), these three accounting for 11.1% (226) of the isolates, while the predominant species of these genera were *Enterobacter cloacae* (64, 41.0%), *Citrobacter freundii* (23, 14.7%), *Enterobacter sakazakii* (14, 9.0%), and *Salmonella Typhimurium* (9, 5.8%).

### 3.3. AST

Susceptibility testing of the predominant isolates (226), that is, *Enterobacter* spp. (171), *Salmonella* spp. (29), and *Citrobacter* spp. (26) to 19 antibiotic agents revealed that two quinolones (NOR, 94.7%, and OFX, 91.2%), a beta-lactamase (IPM, 96%), an aminoglycoside (AK, 95.5%), and chloramphenicol (91.3%) were the most active drugs, while isolates were most resistant to AX (24.7%), AM (21.2%), and AMC (19.9%). FEP however showed a very high rate (48.6%) of intermediate susceptibility.

*Enterobacter* species showed highest resistance to CAZ (38%), AX (76.2%), AM (70.2%), AMC (70.2%), SXT (29.2%), ATM (24%), NA (24.1%), and AZM (25.7%) (Table 6). *Citrobacter* species showed highest resistance to AX (69.2%) and AMC (65.4%). Other drugs to which isolates had increasing resistance were FEP (21.1%), CFM (23.1%), CTX (28%), ATM (23.1%), and NA (23.1%). *Salmonella* were most resistant to AX (69%), AM (65.5%), and AMC (75.9%). Other drugs to which *Salmonella* showed high rates of resistance were AZM (41.4%), CAZ (34.5%), CFM (31%), ATM (31%), CTX (24.1%), and NA (24.1%). No *Salmonella* isolate showed resistance or intermediate susceptibility to OFX (Table 6).

**Figure 2:**
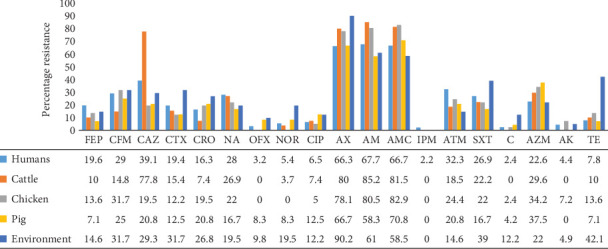
Resistance of non–*E. coli* Enterobacteriaceae by source. Cefepime (FEP), cefixime (CFM), ceftazidime (CAZ), cefotaxime (CTX), ceftriaxone (CRO), nalidixic acid (NA), ofloxacin (OFX), norfloxacin (NOR), ciprofloxacin (CIP), amoxicillin (AX), ampicillin (AM), amoxicillin–clavulanic acid (AMC), imipenem (IPM), aztreonam (ATM), cotrimoxazole (SXT), chloramphenicol C, azithromycin (AZM), amikacin (AK), and tetracycline (TE).

Generally, isolates from all sources had very high resistance rates to AM, AX, and AMC. Resistance to cephalosporins, ATM, SXT, and AZM was also high. Resistance to IPM (2.2%) was observed only among clinical isolates. The highest resistance rates to NOR (19.5%), CIP (12.5%), AX (90.2%), SXT (39%), chloramphenicol (12.2%), and TE (42.1%) were observed among environmental isolates (Figure 2).

**Figure 3:**
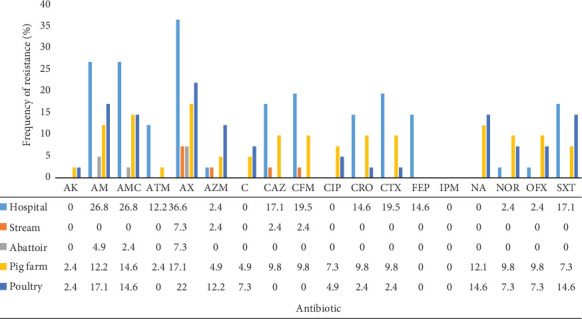
Frequency of resistance of non–*E. coli* Enterobacteriaceae with respect to environmental sources. Cefepime (FEP), cefixime (CFM), ceftazidime (CAZ), cefotaxime (CTX), ceftriaxone (CRO), nalidixic acid (NA), ofloxacin (OFX), norfloxacin (NOR), ciprofloxacin (CIP), amoxicillin (AX), ampicillin (AM), amoxicillin–clavulanic acid (AMC), imipenem (IPM), aztreonam (ATM), cotrimoxazole (SXT), chloramphenicol C, azithromycin (AZM), amikacin (AK), and tetracycline (TE).

With regard to environmental isolates, resistance rates were highest among isolates from the hospital environment where the highest rates were observed for the penicillins and cephalosporins (Figure 3). This was followed by isolates from pig and poultry farms. Resistance was lowest among isolates from abattoir environment.

### 3.4. Antibiotypes

Out of the 226 isolates, 214 (94.7%) showed resistance to one or more antibiotic agents. Resistant strains were grouped into 131 antibiotypes (Supporting Information 1). The predominant antibiotypes were AX^R^ AM^R^ AMC^R^ (25, 11.1%), AX^R^ AM^R^ AMC^R^ AZM^R^ (18, 8.4%), CAZ^R^ AX^R^ AM^R^ AMC^R^ (12, 5.3%), and AX^R^ AMC^R^ AZM^R^ (7, 3.1%). However, most (107, 47.3%) of the antibiotypes were observed only in single isolates. Antibiotype AX^R^ AM^R^ AMC^R^ was detected in *Enterobacter* from chicken, pig, and environment; *Citrobacter* from human and pig; and *Salmonella* from chicken. AX^R^ AM^R^ AMC^R^ AZM^R^ was detected mostly in *Enterobacter* from chicken, human, and environment; *Citrobacter* from pig, and *Salmonella* from human and pig.

Multiresistance (resistance to ≥ 3 antibiotics of different classes) was seen in 54 (25.2%) isolates from various sources and genera (Supporting Information 1). Thirty-four (63.0%), 13 (24.1%), and 7(13.0%) isolates were resistant to 3, 4, and 5 antibiotic classes, respectively. Resistance to five antibiotic classes was observed only in *Enterobacter* spp. and *Citrobacter* spp. isolated from humans, chicken, and the environment (Tables 7 and 8).

### 3.5. Detection of Resistance Genes

#### 3.5.1. PMQR Genes

PMQR genes were detected in 97.8% (44/45). The most prevalent gene was *acrB* (42, 93.3%) followed by the *acrA* (38, 84.4%) and aac(6⁣′)-ib-cr (30, 66.7%), while the least was the *qepA* gene (2, 4.4%) which was detected only in humans (Table 9). The *acrB* gene was detected in isolates from all five sources, with highest detection rate in human 95.5% (21/22). The *acrA* gene was most prevalent 88.6% (31/35) among *Enterobacter* isolates, followed by *Citrobacter* isolates 75% (3/4) and least in *Salmonella* isolates 66.7% (4/6). It was detected in all five sources with the highest occurrence in chicken 100% (4/4%) and environment 100% (5/5). The *aac(6*⁣′* )-ib-cr* was detected in 75% (3/4) of *Citrobacter* isolates, 68.6% (24/35) of *Enterobacter* isolates, and 50% (3/6) of *Salmonella* isolates. It was detected in all five sources with the highest detection rate 81.8% (9/11) in cattle.

#### 3.5.2. The Beta-Lactamase Resistance Genes

The blaCTX-M-1 gene was more frequently detected (14, 22.2%) than the blaCMY-2 gene (5, 7.9%). These genes were detected in isolates from human, cattle, and environmental origin. The blaCTX-M-1 gene was detected in *Enterobacter* and *Salmonella* isolates. It was most prevalent in *Enterobacter* spp. 12/14 (85.7%) when compared to *Salmonella* spp. 2/10 (20%). A similar prevalence was seen in humans 6/14 (42.9%) and cattle 6/14 (42.9%), which was higher when compared to environmental isolates 2/14 (14.3%). The blaCMY-2 gene was detected in *Enterobacter* 4/5 (80.0%) and *Salmonella* spp. 1/5 (20.0%). Among the sources involved, the blaCMY-2 gene was detected only in isolates from humans 3/5 (60.0%) and cattle 2/5 (40.0%), and not the environment.

### 3.6. Cocarriage of Resistance Genes

A total of 80 isolates were involved in the screening of the PMQR and the beta-lactamase genes. Forty-five and 63 of these isolates were tested for PMQR and beta-lactamase genes, respectively, while 28 were tested for both sets. Forty-one (51.3%) isolates carried two or more resistance genes as illustrated in Table 10. Among these, 14 isolates 14/41 (34.1%) carried 2 genes, and 22 (53.7%) carried 3 genes, while 6 (14.6%) carried 4 genes. A total of five (17.9%) isolates of the 28 screened for both the PMQR and the beta-lactamase genes carried both sets of genes.

Of the 14 isolates that carried two genes, most (12, 85.7%) were *Enterobacter* spp., and one isolate each (7.1%) was *Salmonella* and *Citrobacter* spp. With regard to source, the majority were from cattle (5, 35.7%) and chicken (3, 14.4%). For the 22 isolates carrying 3 genes, 17 (77.3%) were *Enterobacter* spp., 3 (13.6%) *Citrobacter* spp., and 2 (9.1%) *Salmonella* spp. The majority (12, 54.5%) were from human and cattle (7, 31.8%). None was observed among chicken isolates. Of the six isolates that carried four genes, most (5, 83.3%) were *Enterobacter* spp., while one (16.7%) was *Salmonella* spp. Most (4, 66.7%) of these isolates were collected from human.

Among the 45 isolates screened for the PMQR genes, 40 (88.9%) carried two or more genes as shown in Table 10. Two isolates (4.4%) were positive for all PMQR genes. These were *Enterobacter* species isolated from human. Twenty-five (55.6%) carried 3 genes each, while 14 (31.1%) carried 2 genes each. Whereas of the 63 isolates screened for the beta-lactamase genes, only 2 (0.6%) carried both genes. These isolates were of the genus *Enterobacter*, isolated from cattle sampled in Mutengene and Limbe. Among the isolates for which both PMQR and beta-lactamase genes were investigated, those positive for any of the beta-lactamase genes were also positive for at least two PMQR genes.

## 4. Discussion

Members of the Enterobacteriaceae family are zoonotic and cause diseases such as urinary tract infection, abscesses, septicaemia, enteric fever, gastroenteritis, and meningitis in humans and animals [32]. These organisms being part of the bacterial commensals of food animals remain a potential threat to mankind as they constantly spread from these sources to the environment [4]. Even though the infections they cause are usually self-limiting in immunocompetent humans, they cause serious health problems to immunocompromised persons requiring prompt treatment with effective antibiotics. Carbapenem-resistant strains have emerged lately and have devastating consequences on both human and veterinary heath including the healthy population [33].

In this study, non–*E. coli* Enterobacteriaceae made up 12.7% (260/2042) of all bacteria isolated from the different sources. The species of *Enterobacter* (171, 65.8%), *Salmonella* (29, 11.2%), and *Citrobacter* (26, 10.0%) genera were most frequently isolated. This low general prevalence could be explained by the fact that the gut of mammals is dominated by other bacterial species [34]. The relatively high prevalence of *Enterobacter* spp. and low prevalence of *Citrobacter* spp. did not agree with the results obtained by Otote et al. in Nigeria where *Citrobacter* spp. were more frequently isolated than *Enterobacter* spp. in clinical specimens [35]. No significant difference was seen in the distribution of these isolates across the various sources (Table 5), implying the use of whole stool, rectal swabbing, and swabbing environmental objects are all reliable techniques for the laboratory isolation of Enterobacteriaceae.

Antibiogram of predominant isolates revealed that the most active drugs were the quinolones (NOR and OFX), a beta-lactamase (IPM), an aminoglycoside (AK), and chloramphenicol. Isolates showed highest resistance to penicillins (AX, AM, and AMC). These results corroborate the findings of Leinyuy et al. who reported a marked susceptibility of Enterobacteriaceae isolated from chicken to IPM, AK, a reduced susceptibility to cephalosporins (CRO and CTX), and a marked resistance to penicillin (AX and AMC) [10]. The high resistance seen with penicillins could partly be due to the natural resistance exhibited by *Enterobacter* isolates against the latter [36]. TE is the most consumed antibiotic in the animal-rearing sector in Cameroon [37]. The overall susceptibility to this antibiotic of 80.2% could be an indication of good stewardship in both clinical and veterinary practice. The very high intermediate resistance rate to FEP (a fourth-generation cephalosporin) is a great call for concern as this indicates a shift towards resistance. It is challenging to explain this phenomenon given that it is a relatively new drug and does not exist in oral form, thus discouraging abusive use. However, it was observed that environmental isolates showed a leading resistance to more antibiotics when compared to those of the other sources. This was consistent with the findings of Tesfaye et al. [38]. This high resistance rate from environmental isolates could be a result of the high-frequency exchange of mobile genetic elements such as plasmids among bacterial isolates from diverse sources [39]. Although chloramphenicol showed an impressively high activity (91.3%) against the isolates, its use on both humans and food animals is discouraged in some countries, including Cameroon. This is owing to its known severe adverse effects, such as bone marrow toxicity and grey baby syndrome [40].

In this study, we observed a higher carriage of PMQR genes than beta-lactamase genes with the frequency of occurrence of the ESBL gene (*blaCTX-M-1*) higher than that of the ampC gene (*blaCMY-2*). Also, the frequency of carriage of *acrA* and *acrB* was markedly higher and similar compared to the other PMQR genes screened. This could be because they belong to the same family of efflux systems, the resistance-nodulation-cell division (RND) family that catalyze substrate efflux via a substrate/H+ antiport mechanism and are found in numerous gram-negative bacteria [41–43]. However, we observed that the general higher activity seen with quinolones compared to beta-lactams (cephalosporins) was in contrast with the frequency of detection of each set of the corresponding resistance genes 97.8% (44/45) for PMQR and 30.1% (19/63) for beta-lactamase genes. This suggests that these plasmid-borne quinolone resistance genes screened may not be very potent, while the resistance seen with beta-lactams stems from some other determinants. There was generally high-level cocarriage of resistance genes among isolates, most of them belonging to the genus *Enterobacter* particularly those of human origin (Table 10). This highlights the need for continuous surveillance of these genes as the efficacies of the quinolone and beta-lactamase antibiotics are at stake. Multiresistance (resistance to ≥ 3 antibiotics of different classes) was seen in 54 (25.2%) isolates. This is a further indication that several antibiotics are losing efficacy against members of the Enterobacteriaceae family. Most of the multiresistant strains were from humans (21, 38.9%) followed by the environment (13, 24.1%) and least from pigs (3, 5.6%). This suggests that the degree of antibiotic abuse is higher among humans when compared to the other sources.

A total of 131 antibiotypes were seen from the isolates with AX^R^ AM^R^ AMC^R^ (25, 11.1%), AX^R^ AM^R^ AMC^R^ AZM^R^ (18, 8.4%), CAZ^R^ AX^R^ AM^R^ AMC^R^ (12, 5.3%), and AX^R^ AMC^R^ AZM^R^ (7, 3.1%), being the most common among the predominant Enterobacteriaceae and from the different sources. This could imply that the same strains circulate among humans, food animals, and the environment, most probably by means of cross-contamination. Also, strains of different genera having the same resistance patterns could imply the possibility of the exchange of genetic material such as plasmids [39] among them. Our finding show high levels of multidrug-resistant non–*E. coli* Enterobacteriaceae and high-level circulation of resistant strains between humans, animals, and the environment and the exchange of resistance markers, highlighting the potential risk of these strains becoming established in the Fako Division. This underscores the need for constant monitoring of the resistance patterns of these organisms in Fako.

## 5. Limitations

Microbial source tracking was based on antibiotype homology. Nucleic acid–based techniques such as multilocus sequence typing would be more reliable. The study could not extend to Muyuka subdivision due to insecurity linked to the sociopolitical crisis at the time of sample collection. This division is part of the Fako Division, and so the findings of this study may not be very accurate if generalised over the entire division. Positive control organisms were not available for resistance gene detection PCR experiments.

## 6. Conclusion

This study provides evidence of the buildup of antibiotic resistance among non–*E. coli* members of the Enterobacteriaceae family from humans, chickens, pigs, cattle, and the environment. The findings further predict the circulation of these bacterial species among the different sources investigated, hence the urgent need for the putting in place of an effective surveillance programme from a one-health perspective, which reports antimicrobial resistance patterns of these organisms circulating for appropriate government action.

## Figures and Tables

**Table 1:** Polymerase chain reaction conditions and primer sequences used for the identification of non–*E. coli* Enterobacteriaceae.

	**Organism**	**Target gene**	**Primer sequence**	**PCR cycling parameters**	**Amplicon size (bp)**	**Reference**
**Genus-specific amplification**						
1	*Salmonella* serotypes	*16S rDNA*	F: GTGTTGTGGTTAATAACCGCAGCA (16S-Sal) R: TGTTBGMTCCCCACGCTTTCG (16S-CCR) NB: B represents G, C, or T; M represents A or C	Initial denaturation: 95°C for 5 min Denaturation: 94°C for 20 s Annealing: 66°C for 30 s Extension: 72°C for 30 s Final extension: 72°C for 5 min Number of PCR cycles: 35 Hold: 4°C Agarose: 2%	324	Lin et al., [23]

2	*Citrobacter* spp.	*16S rRNA*	F: 5⁣′-GCT CAA CCT GGG AAC TGC ATC CGA-3⁣′ R: 5⁣′AGT TCC GGC CTA ACC GCT GGC AA-3⁣′	Initial denaturation: 95°C for 10 min Denaturation: 94°C for 45 s Annealing: 58°C for 45 s Extension: 72°C for 90 s Final extension: 72°C for 10 min Number of PCR cycles: 35 Hold: 12°C Agarose: 2%	529	Anbazhagan et al., [24]

3	*Enterobacter* spp.	*dnaJ*	F: 5⁣′-GACCTGCGCTACAACATGGAKCT-3⁣′ R: 5⁣′-CCGCGYTCCAAAAGCTTCTTYGAT-3⁣′	Initial denaturation: 94°C for 4 min Denaturation: 94°C for 50 s Annealing: 60°C for 35 s Extension: 72°C for 60 s Final extension: 72°C for 5 min Number of PCR cycles: 35 Agarose: 1.5%	714 bp	Hernandez-Alonso, [25]

**Table 2:** Polymerase chain reaction conditions and specific primers for the screening of resistance genes.

	**Target gene**	**Primer sequence**	**PCR cycling parameters**	**Expected band size (bp)**	**Reference**
Beta-lactamase genes					

1	*blaCTX-M-1* (ESBL)	F: AAC CGT CAC GCT GTT GTT AG R: TTG AGG CTG GGT GAA GTA AG	Initial denaturation: 95°C for 10 min Denaturation: 95°C for 30 s Annealing: 55 for 60 s Extension: 72°C for 60 s Final extension: 72°C for 7 min Number of PCR cycles: 30 Hold: 4°C	766	[30]
2	*blaCMY-2* (AmpC)	F: 5⁣′TGG CCG TTG CCG TTA TCT AC3⁣′ R: 5⁣′CCC GTT TTA TGC ACC CAT GA3⁣′	Initial denaturation: 95°C for 10 min Denaturation: 95°C for 30 s Annealing: 55 for 60 s Extension: 72°C for 60 s Final extension: 72°C for 7 min Number of PCR cycles: 30 Hold: 4°C	870	[30]

Quinolone genes					

3	*qepA*	F: 5⁣′CTGCAGGTACTGCGTCATG3⁣′ R: 5⁣′CGTGTTGCTGGAGTTCTTC3⁣′	Initial denaturation: 94°C for 5 min Denaturation: 94°C for 45 s Annealing: 51 for 45 s Extension: 72°C for 45 s Final extension: 72°C for 5 min Number of PCR cycles: 36 Hold: 4°C	403	[31]
4	*acrA*	F: 5⁣′TCTGATCGACGGTGACATCC3⁣′ R: 5⁣′TCGAGCAATGATTTCCTGCG3⁣′	Initial denaturation: 94°C for 5 min Denaturation: 94°C for 45 s Annealing: 57 for 45 s Extension: 72°C for 45 s Final extension: 72°C for 5 min Number of PCR cycles: 36 Hold: 4°C	157	[31]
5	*acrB*	F: 5⁣′CAATACGGAAGAGTTTGGCA3⁣′ R: 5⁣′CAGACGAACCTGGGAACC3⁣′	Initial denaturation: 94°C for 5 min Denaturation: 94°C for 45 s Annealing: 52 for 45 s Extension: 72°C for 45 s Final extension: 72°C for 5 min Number of PCR cycles: 36 Hold: 4°C	64	[31]

Aminoglycoside and fluoroquinolone gene					

6	*aac(6*⁣′*)-ib-cr*	F: 5⁣′TTGCGATGCTCTATGAGTGGCTA R: 5⁣′CTCGAATGCCTGGCGTGTTT	Initial denaturation: 94°C for 5 min Denaturation: 94°C for 45 s Annealing: 55 for 45 s Extension: 72°C for 45 s Final extension: 72°C for 5 min Number of PCR cycles: 36 Hold: 4°C	611	[31]

**Table 3:** Distribution of samples collected by source and by locality.

**Locality**	**Source**					
	**Human** **n** **(%)**	**Chicken** **n** ** (%)**	**Pig** **n** ** (%)**	**Cow** **n** ** (%)**	**Environment** **n** ** (%)**	**Total** **n** ** (%)**
Buea	186 (9.1)	112 (5.5)	42 (2.1)	86 (4.2)	135 (6.6)	571 (28.0)
Mutengene	282 (13.8)	60 (2.9)	12 (0.6)	33 (1.6)	36 (1.8)	423 (20.7)
Tiko	338 (16.6)	186 (9.1)	35 (1.7)	0 (00.0)	62 (3.0)	621 (30.4)
Limbe	170 (8.3)	81 (4.0)	57 (2.8)	29 (1.4)	90 (4.4)	427 (20.9)
Total	976 (47.8)	449 (22)	146 (7.1)	148 (7.2)	323 (15.8)	2042 (100.0)

*Note: n* = number of isolates.

**Table 4:** Distribution of environmental samples with respect to source and locality.

**Locality**	**Source**					
	**Hospital** **n** ** (%)**	**Stream** **n** ** (%)**	**Slaughterhouse** **n** ** (%)**	**Pig farm** **n** ** (%)**	**Poultry** **n** ** (%)**	**Total** **n** ** (%)**
Limbe	19 (5.9)	13 (4.0)	15 (4.6)	17 (5.3)	18 (5.6)	82 (25.4)
Buea	19 (5.9)	23 (7.1)	19 (5.9)	15 (4.6)	19 (5.9)	95 (29.4)
Mutengene	8 (2.5)	14 (4.3)	16 (5.0)	19 (5.9)	15 (4.6)	72 (22.3)
Tiko	12 (3.7)	20 (6.2)	0 (0.0)	21 (6.5)	21 (6.5)	74 (22.9)
Total	58 (18.0)	70 (21.7)	50 (15.5)	72 (22.3)	73 (22.6)	323 (100.0)

*Note: p* = 0.93; *n* = number of isolates.

**Table 5:** Non–*E. coli* Enterobacteriaceae genera identified.

**Genus**	**Source**					**Total** **n** ** (%)**
	**Human** **n** ** (%)**	**Chicken** **n** ** (%)**	**Pig** **n** ** (%)**	**Cow** **n** ** (%)**	**Environment** **n** ** (%)**	
*Cedecea* spp.	0 (0.0)	0 (0.0)	0 (0.0)	0 (0.0)	1 (0.4)	1 (0.4)
*Chryseomonas* spp.	1 (0.4)	0 (0.0)	0 (0.0)	0 (0.0)	0 (0.0)	1 (0.4)
***Citrobacter* spp.**	13 (5.0)	3 (1.2)	7 (2.7)	0 (0.0)	2 (0.8)	**26 (10.0)**
***Enterobacter* spp.**	68 (26.1)	31 (11.9)	11 (4.2)	24 (9.2)	37 (14.2)	**171 (65.8)**
*Escherichia* spp.	0 (0.0)	0 (0.0)	0 (0.0)	0 (0.0)	3 (1.2)	3 (1.1)
*Hafnia* spp.	0 (0.0)	0 (0.0)	0 (0.0)	0 (0.0)	2 (0.8)	2 (0.8)
*Klebsiella* spp.	2 (0.8)	0 (0.0)	0 (0.0)	0 (0.0)	3 (1.2)	6 (2.3)
*Morganella* spp.	0 (0.0)	0 (0.0)	0 (0.0)	0 (0.0)	1 (0.4)	1 (0.4)
*Pantoea* spp.	0 (0.0)	0 (0.0)	0 (0.0)	0 (0.0)	6 (2.3)	6 (2.3)
*Proteus* spp.	0 (0.0)	3 (1.2)	0 (0.0)	0 (0.0)	2 (0.8)	6 (2.3)
*Providencia* spp.	1 (0.4)	0 (0.0)	0 (0.0)	0 (0.0)	0 (0.0)	1 (0.4)
*Rahnella* spp.	0 (0.0)	0 (0.0)	0 (0.0)	0 (0.0)	2 (0.8)	2 (0.8)
*Raoultella* spp.	0 (0.0)	0 (0.0)	0 (0.0)	0 (0.0)	1 (0.4)	1 (0.4)
***Salmonella* spp.**	12 (4.6)	6 (2.3)	6 (2.3)	3 (1.2)	2 (0.8)	**29 (11.2)**
*Serratia* spp.	0 (0.0)	0 (0.0)	0 (0.0)	0 (0.0)	3 (1.2)	13 (5.0)
*Tatumella* spp.	0 (0.0)	1 (0.4)	0 (0.0)	0 (0.0)	0 (0.0)	1 (0.4)
Total	97 (37.3)	46 (17.7)	24 (9.2)	27 (10.4)	66 (25.4)	260 (100)

*Note: *Genus names in bold were the predominant ones. These were the only genera involved in downstream experiments. *p* = 0.7; *n* = number of isolates.

**Table 6:** General antibiotic susceptibility pattern of the predominant non–*E. coli* Enterobacteriaceae.

**Antibiotic**	***Enterobacter* spp.**				***Citrobacter* spp.**				***Salmonella* spp.**				**Cum S**	**Cum I**	**Cum R**
	**n** ** S (%)**	**n** ** I (%)**	**n** ** R (%)**	**Total**	**n** ** S (%)**	**n** ** I (%)**	**n** ** R (%)**	**Total**	**n** ** S (%)**	**n** ** I (%)**	**n** ** R (%)**	**Total**	**n** ** (%)**	**n** ** (%)**	**n** ** (%)**
Cefepime (FEP)	43 (41.0)	45 (42.9)	17 (16.2)	105	2 (10.5)	13 (68.4)	4 (21.1)	19	5 (35.7)	9 (64.3)	0 (0.0)	14	50 (36.2)	67 (48.6)	21 (15.2)
Cefixime (CFM)	100 (58.5)	23 (13.5)	48 (28.1)	171	16 (61.5)	4 (15.4)	6 (23.1)	26	16 (55.2)	4 (13.8)	9 (31.0)	29	132 (58.5)	31 (13.7)	63 (27.9)
Ceftazidime (CAZ)	92 (53.8)	14 (8.2)	65 (38.0)	171	16 (64.0)	2 (8.0)	7 (28.0)	25	13 (44.8)	6 (20.7)	10 (34.5)	29	121 (53.8)	22 (9.8)	82 (36.4)
Cefotaxime (CTX)	122 (71.8)	16 (9.4)	32 (18.8)	170	19 (73.1)	3 (11.5)	4 (15.4)	26	18 (62.1)	4 (13.8)	7 (24.1)	29	159 (70.7)	23 (10.2)	43 (19.1)
Ceftriaxone (CRO)	123 (71.9)	18 (10.5)	30 (17.5)	171	19 (76.0)	1 (4.0)	5 (20.0)	25	21 (72.4)	2 (6.9)	6 (20.7)	29	163 (72.4)	21 (9.3)	41 (18.2)
Amoxicillin (AX)	39 (23.2)	1 (0.6)	128 (76.2)	168	7 (26.9)	1 (3.9)	18 (69.2)	26	9 (31.0)	0 (0.0)	20 (69.0)	29	55 (24.7)	2 (0.9)	166 (74.4)
Ampicillin (AM)	34 (19.9)	17 (9.9)	120 (70.2)	171	7 (26.9)	0 (0.0)	19 (73.1)	26	7 (24.1)	3 (10.3)	19 (65.5)	29	48 (21.2)	20 (8.9)	158 (69.9)
Amoxiclav (AMC)	33 (19.3)	18 (10.5)	120 (70.2)	171	7 (26.9)	2 (7.7)	17 (65.4)	26	5 (17.2)	2 (6.9)	22 (75.9)	29	45 (19.9)	22 (9.7)	159 (70.4)
Imipenem (IPM)	163 (95.3)	7 (4.1)	1 (0.6)	171	25 (96.2)	0 (0.0)	1 (3.9)	26	29 (100.0)	0 (0.0)	0 (0.0)	29	217 (96.0)	7 (3.1)	2 (0.9)
Aztreonam (ATM)	126 (73.7)	4 (2.3)	41 (24.0)	171	19 (73.1)	1 (3.9)	6 (23.1)	26	20 (69.0)	0 (0.0)	9 (31.0)	29	165 (73.0)	5 (2.2)	56 (24.8)
Nalidixic acid (NA)	114 (67.1)	15 (8.8)	41 (24.1)	170	17 (65.4)	3 (11.5)	6 (23.1)	26	21 (72.4)	1 (3.5)	7 (24.1)	29	152 (67.6)	19 (8.4)	54 (24.0)
Ofloxacin (OFX)	154 (90.1)	2 (1.2)	15 (8.8)	171	25 (96.2)	1 (3.9)	0 (0.0)	26	27 (93.1)	1 (3.5)	1 (3.5)	29	206 (91.2)	4 (1.8)	16 (7.1)
Norfloxacin (NOR)	160 (93.6)	3 (1.8)	8 (4.7)	171	25 (96.2)	0 (0.0)	1 (3.9)	26	29 (100.0)	0 (0.0)	0 (0.0)	29	214 (94.7)	3 (1.3)	9 (4.0)
Ciprofloxacin (CIP)	135 (79.9)	19 (11.2)	15 (8.9)	169	20 (76.9)	4 (15.4)	2 (7.7)	26	25 (86.2)	3 (10.3)	1 (3.5)	29	180 (80.4)	26 (11.6)	18 (8.0)
Cotrimoxazole (SXT)	117 (68.4)	4 (2.3)	50 (29.2)	171	21 (80.8)	0 (0.0)	5 (19.2)	26	24 (82.8)	0 (0.0)	5 (17.2)	29	162 (71.7)	4 (1.8)	60 (26.6)
Chloramphenicol C	142 (91.0)	8 (5.1)	6 (3.9)	156	23 (95.8)	1 (0.0)	1 (4.2)	25	24 (88.9)	1 (3.7)	2 (7.4)	27	189 (91.3)	9 (4.4)	9 (4.4)
Azithromycin (AZM)	127 (74.3)	0 (0.0)	44 (25.7)	171	21 (80.8)	0 (0.0)	5 (19.2)	26	17 (58.6)	0 (0.0)	12 (41.4)	29	165 (73.0)	0 (0.0)	61 (27.0)
Amikacin (AK)	163 (95.9)	0 (0.0)	7 (4.1)	170	24 (96.0)	0 (0.0)	1 (4.0)	25	27 (93.1)	1 (3.5)	1 (3.5)	29	214 (95.5)	1 (0.45)	9 (4.0)
Tetracycline (TE)	69 (79.3)	4 (4.6)	14 (16.1)	87	13 (76.5)	2 (11.8)	2 (11.8)	17	11 (91.7)	0 (0.0)	1 (8.3)	12	93 (80.2)	6 (5.2)	17 (14.7)

*Note: n* = number of isolates.

Abbreviations: S = sensitive, I = intermediate, R = resistant.

**Table 7:** Distribution of multidrug-resistant non–*E. coli* Enterobacteriaceae by genus.

**No. of antibiotic classes**	**Genera**			
	***Enterobacter* spp.** **n** ** (%)**	***Citrobacter* spp.** **n** ** (%)**	***Salmonella* spp.** **n** ** (%)**	**Total** **n** ** (%)**
3	25 (46.3)	2 (3.7)	7 (13.0)	34 (63.0)
4	11 (20.4)	1 (1.9)	1 (1.9)	13 (24.1)
5	6 (11.1)	1 (1.9)	0 (0.0)	7 (13.0)
Total	42 (77.8)	4 (7.4)	8 (14.8)	54

*Note: p* = 0.08; *n* = number of isolates.

**Table 8:** Distribution of multidrug-resistant non–*E. coli* Enterobacteriaceae by source.

**No. of antibiotic classes**	**Source**					
	**Human** **n** ** (%)**	**Chicken** **n** ** (%)**	**Pig** **n** ** (%)**	**Cattle** **n** ** (%)**	**Env.** **n** ** (%)**	**Total** **n** ** (%)**
3	15 (27.8)	5 (9.3)	2 (3.7)	6 (11.1)	6 (11.1)	34 (63.0)
4	4 (7.4)	3 (5.6)	1 (1.9)	2 (3.7)	3 (5.6)	13 (21.1)
5	2 (3.7)	1 (1.9)	0 (0.0)	0 (0.0)	4 (7.4)	7 (13.0)
Total	21 (38.9)	9 (16.7)	3 (5.6)	8 (14.8)	13 (24.1)	54

*Note: p* = 0.39; Env. = environment; *n* = number of isolates.

**Table 9:** Prevalence of plasmid-borne resistance genes in non*–E. coli* Enterobacteriaceae.

**Source**	**Genus**	**PMQR genes, ** **N** = 45					**bla genes, ** **N** = 63		
		**aac(6**⁣′**)-ib-cr** **n (%)**	**qepA** **n** ** (%)**	**acrA** **n** ** (%)**	**acrB** **n** ** (%)**	**Total**	**blaCTX-M-1** **n** ** (%)**	**blaCMY-2** **n** ** (%)**	**Total**
Human	*Enterobacter*	10 (22.2)	2 (4.4)	13 (28.9)	14 (31.1)	39	4 (6.3)	2 (3.2)	6
	*Salmonella*	3 (6.7)	0 (00.0)	3 (6.7)	4 (8.9)	10	2 (3.2)	1 (1.6)	3
	*Citrobacter*	3 (6.7)	0 (00.0)	2 (4.4)	3 (6.7)	8	0 (00.0)	0 (00.0)	0

Chicken	*Enterobacter*	1 (2.2)	0 (00.0)	3 (6.7)	2 (4.4)	6	0 (00.0)	0 (00.0)	0
	*Salmonella*	NT	NT	NT	NT	/	NT	NT	/
	*Citrobacter*	0 (0.0)	0 (00.0)	1 (2.2)	1 (2.2)	2	0 (00.0)	0 (00.0)	0

Pig	*Enterobacter*	2 (4.4)	0 (00.0)	1 (2.20)	1 (2.2)	4	0 (00.0)	0 (00.0)	0
	*Salmonella*	0 (00.0)	0 (00.0)	0 (00.0)	1 (2.2)	1	0 (00.0)	0 (00.0)	0
	*Citrobacter*	NT	NT	NT	NT	/	NT	NT	/

Cattle	*Enterobacter*	9 (20)	0 (00.0)	9 (20)	10 (22.2)	28	6 (9.5)	2 (3.2)	8
	*Salmonella*	0 (0.0)	0 (00.0)	1 (2.2)	1 (2.2)	2	0 (00.0)	0 (00.0)	0
	*Citrobacter*	NT	NT	NT	NT	/	NT	NT	/

Environment	*Enterobacter*	2 (4.4)	0 (00.0)	5 (11.1)	5 (11.1)	12	2 (3.2)	0 (00.0)	2
	*Salmonella*	0 (00.0)	0 (00.0)	0 (00.0)	0 (00.0)	0	0 (00.0)	0 (00.0)	0
	*Citrobacter*	NT	NT	NT	NT	/	NT	NT	/

Total		30 (66.7)	2 (4.4)	38 (84.4)	42 (93.3)		14 (22.2)	5 (7.9)	

*Note: p* = 0.03.

Abbreviation: NT = not tested.

**Table 10:** Cocarriage of PMQR and beta-lactamase genes by non–*E. coli* Enterobacteriaceae.

**SN**	**Source**	**Genus**	***qepA***	***acrA***	***acrB***	***aac(6*** ⁣′***)-ib-cr***	***blaCMY-2***	***blaCTX-M-1***	**No. of genes positive**
1	Human	*Enterobacter*	−	+	+	+	NT	NT	3
2	Human	*Salmonella*	−	+	+	+	−	−	3
3	Human	*Enterobacter*	−	+	+	+	−	+	4
4	Human	*Citrobacter*	−	+	+	+	−	−	3
5	Human	*Citrobacter*	−	+	+	+	NT	NT	3
6	Human	*Enterobacter*	−	+	+	+	−	−	3
7	Human	*Enterobacter*	−	+	+	+	−	−	3
8	Human	*Enterobacter*	−	−	−	−	−	−	0
9	Chicken	*Enterobacter*	−	+	−	+	NT	NT	2
10	Human	*Enterobacter*	−	+	+	−	NT	NT	2
11	Human	*Enterobacter*	−	+	+	+	−	−	3
12	Chicken	*Enterobacter*	−	+	+	−	NT	NT	2
13	Human	*Enterobacter*	+	+	+	+	−	−	4
14	Human	*Salmonella*	−	+	+	+	−	−	3
15	Human	*Salmonella*	−	+	+	+	+	−	4
16	Chicken	*Citrobacter*	−	+	+	−	NT	NT	2
17	Human	*Citrobacter*	−	−	+	+	−	−	3
18	Human	*Salmonella*	−	−	+	−	−	−	1
19	Human	*Enterobacter*	+	+	+	+	−	−	4
20	Human	*Enterobacter*	−	+	+	+	−	−	3
21	Pig	*Salmonella*	−	−	+	−	NT	NT	1
22	Human	*Enterobacter*	−	−	+	−	−	−	1
23	Cattle	*Enterobacter*	−	+	+	+	−	−	3
24	Cattle	*Enterobacter*	−	+	+	+	−	−	3
25	Cattle	*Salmonella*	−	+	+	−	−	−	2
26	Cattle	*Enterobacter*	−	+	+	+	NT	NT	3
27	Cattle	*Enterobacter*	−	+	+	+	−	−	3
28	Pig	*Enterobacter*	−	+	+	+	NT	NT	3
29	Cattle	*Enterobacter*	−	+	+	+	−	+	4
30	Cattle	*Enterobacter*	−	+	+	+	−	+	4
31	Cattle	*Enterobacter*	−	+	+	+	−	−	3
32	Cattle	*Enterobacter*	−	−	+	+	−	−	2
33	Cattle	*Enterobacter*	−	+	+	−	−	−	2
34	Cattle	*Enterobacter*	−	+	+	+	NT	NT	3
35	Human	*Enterobacter*	−	+	+	+	−	−	3
36	Human	*Enterobacter*	−	+	+	−	NT	NT	2
37	Human	*Enterobacter*	−	+	+	+	−	−	3
38	Human	*Enterobacter*	−	+	+	−	−	−	2
39	Environment	*Enterobacter*	−	+	+	−	NT	NT	2
40	Chicken	*Enterobacter*	−	+	+	−	NT	NT	2
41	Environment	*Enterobacter*	−	+	+	+	NT	NT	3
42	Pig	*Enterobacter*	−	−	−	+	NT	NT	1
43	Environment	*Enterobacter*	−	+	+	−	NT	NT	2
44	Environment	*Enterobacter*	−	+	+	−	−	+	3
45	Environment	*Enterobacter*	−	+	+	+	NT	NT	3
46	Human	Enterobacter	NT	NT	NT	NT	−	−	0
47	Human	Enterobacter	NT	NT	NT	NT	−	−	0
48	Human	Enterobacter	NT	NT	NT	NT	−	−	0
49	Human	Enterobacter	NT	NT	NT	NT	−	+	1
50	Human	Salmonella	NT	NT	NT	NT	−	−	0
51	Human	Citrobacter	NT	NT	NT	NT	−	−	0
52	Human	Enterobacter	NT	NT	NT	NT	−	−	0
53	Human	Enterobacter	NT	NT	NT	NT	−	−	0
54	Human	Enterobacter	NT	NT	NT	NT	−	−	0
55	Human	Enterobacter	NT	NT	NT	NT	−	−	0
56	Human	Enterobacter	NT	NT	NT	NT	−	+	1
57	Human	Enterobacter	NT	NT	NT	NT	−	−	0
58	Human	Enterobacter	NT	NT	NT	NT	−	−	0
59	Human	Salmonella	NT	NT	NT	NT	−	+	1
60	Cattle	Enterobacter	NT	NT	NT	NT	−	−	0
61	Cattle	Enterobacter	NT	NT	NT	NT	−	−	0
62	Cattle	Enterobacter	NT	NT	NT	NT	−	+	1
63	Cattle	Enterobacter	NT	NT	NT	NT	−	−	0
64	Cattle	Enterobacter	NT	NT	NT	NT	−	−	0
65	Cattle	Enterobacter	NT	NT	NT	NT	+	+	2
66	Cattle	Enterobacter	NT	NT	NT	NT	−	+	1
67	Cattle	Salmonella	NT	NT	NT	NT	−	−	0
68	Cattle	Enterobacter	NT	NT	NT	NT	−	−	0
69	Cattle	Enterobacter	NT	NT	NT	NT	+	+	2
70	Human	Enterobacter	NT	NT	NT	NT	−	+	1
71	Human	Salmonella	NT	NT	NT	NT	−	+	1
72	Human	Enterobacter	NT	NT	NT	NT	+	−	1
73	Human	Enterobacter	NT	NT	NT	NT	+	−	1
74	Environment	Enterobacter	NT	NT	NT	NT	−	−	0
75	Environment	Enterobacter	NT	NT	NT	NT	−	−	0
76	Environment	Enterobacter	NT	NT	NT	NT	−	−	0
77	Environment	Enterobacter	NT	NT	NT	NT	−	+	1
78	Environment	Salmonella	NT	NT	NT	NT	−	−	0
79	Environment	Enterobacter	NT	NT	NT	NT	−	−	0
80	Environment	Enterobacter	NT	NT	NT	NT	−	−	0

Abbreviation: NT = not tested.

## Data Availability

All data obtained during this study is available and can be shared for relevant purpose.

## Nomenclature

FEP: cefepime
CFM: cefixime
CAZ: ceftazidime
CTX: cefotaxime
CRO: ceftriaxone
NA: nalidixic acid
OFX: ofloxacin
NOR: norfloxacin
CIP: ciprofloxacin
AX: amoxicillin
AM: ampicillin
AMC: amoxicillin–clavulanic acid
IPM: imipenem
ATM: aztreonam
SXT: cotrimoxazole
AZM: chloramphenicol C, azithromycin
AK: amikacin
TE: tetracycline
PMQR genes: plasmid-mediated quinolone resistance
bla: beta-lactamase
CLED: cysteine lactose electrolyte deficient agar
API 20E: analytical profile index for Enterobacteriaceae
PCR: polymerase chain reaction
CLSI: Clinical and Laboratory Standards Institute
BSAC: British Society for Antimicrobial Chemotherapy

## Supporting Information

Supporting Information 1 The PCR gel image of the acrA antibiotic resistance gene.

Supporting Information 2 The PCR gel image of the acrB antibiotic resistance gene.

Supporting Information 3 AST raw data containing diameter of zone of inhibition.

Supporting Information 4 The PCR gel image of the blaCMY-2 antibiotic resistance gene.

Supporting Information 5 The PCR gel image of the blaCTX-M-1 antibiotic resistance gene.

Supporting Information 6 The PCR gel image of the *16SrRNA* gene used to confirm the identity of isolates of the *Citrobacter* genus.

Supporting Information 7 The PCR gel image of the 16S rDNA gene used to confirm the identity of isolates of the *Salmonella* genus.

Supporting Information 8 A table that summarises the different antibiotypes found in our study.
