# Supplementary figures and images for: Resistance Profiling of Predominant Non–E. coli Enterobacteriaceae Isolated From Humans, Food Animals, and the Environment in the Fako Division of Cameroon
Source: Biomed Res Int. 2025 Jun 23;2025:3947539. doi: 10.1155/bmri/3947539 (PMC12208769; doi:10.1155/bmri/3947539)

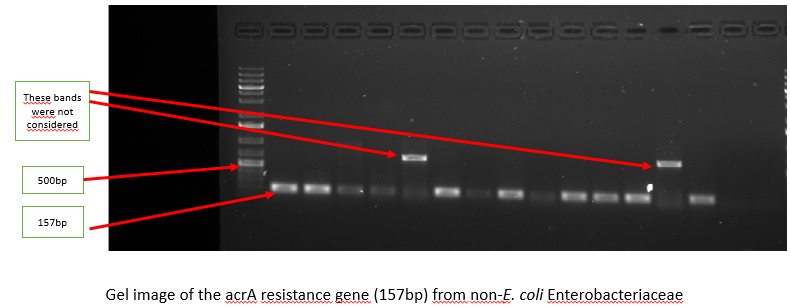

Supplement: Supporting Information 1 — The PCR gel image of the acrA antibiotic resistance gene. [file 3947539.f1.png]

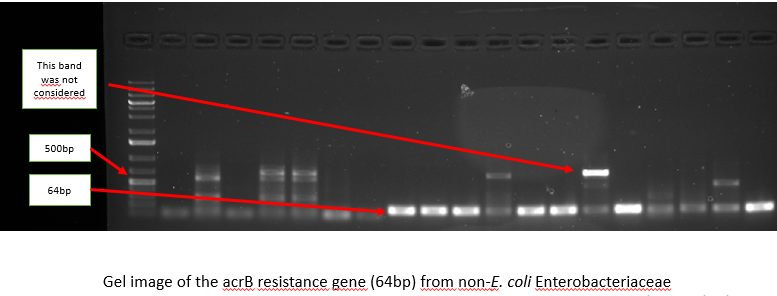

Supplement: Supporting Information 2 — The PCR gel image of the acrB antibiotic resistance gene. [file 3947539.f2.png]

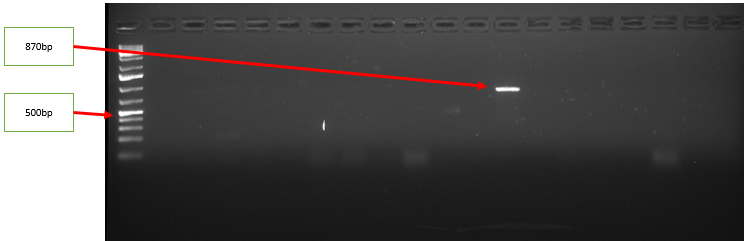

Supplement: Supporting Information 4 — The PCR gel image of the blaCMY-2 antibiotic resistance gene. [file 3947539.f4.png]

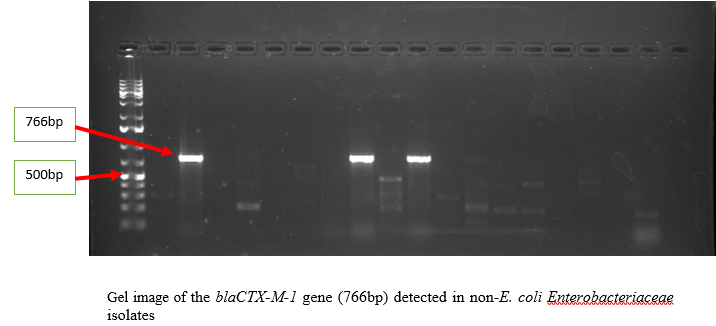

Supplement: Supporting Information 5 — The PCR gel image of the blaCTX-M-1 antibiotic resistance gene. [file 3947539.f5.png]

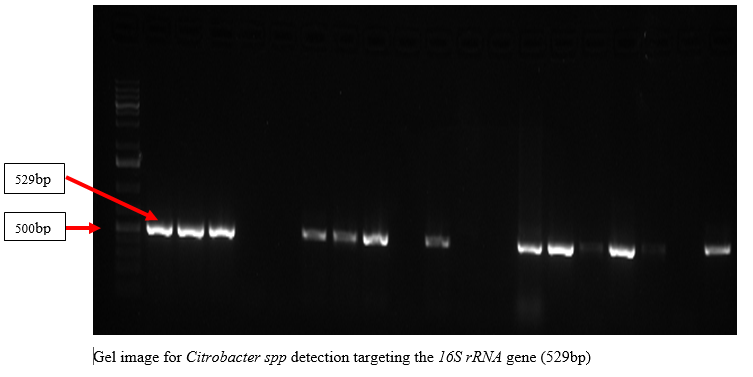

Supplement: Supporting Information 6 — The PCR gel image of the 16SrRNA gene used to confirm the identity of isolates of the Citrobacter genus. [file 3947539.f6.png]

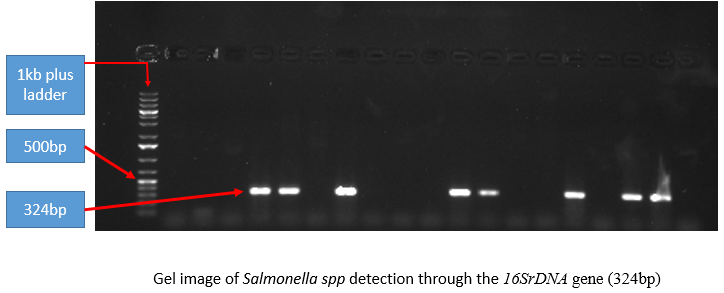

Supplement: Supporting Information 7 — The PCR gel image of the 16S rDNA gene used to confirm the identity of isolates of the Salmonella genus. [file 3947539.f7.png]
