# Supplementary material for: Resistance Profiling of Predominant Non–E. coli Enterobacteriaceae Isolated From Humans, Food Animals, and the Environment in the Fako Division of Cameroon
Source: Biomed Res Int. 2025 Jun 23;2025:3947539. doi: 10.1155/bmri/3947539 (PMC12208769; doi:10.1155/bmri/3947539)
Supplement: Supporting Information 8 — A table that summarises the different antibiotypes found in our study. [file 3947539.f8.docx]

**Supplementary file 1**: Distribution of antibiotypes with respect to genus and source

| NO | Antibiotype | Genus | Source | No of Strains (%) | No of Classes |
| --- | --- | --- | --- | --- | --- |
|  | Resistance to single antibiotic agent |  |  | 9 |  |
| A1.1 | FEP^R^ | Cit | Human | 1(0.4) | 1 |
| A1.2 | CTX^R^ | Ent | Human | 1(0.4) | 1 |
| A1.5 | AMC^R^ | Ent | Chicken | 2(0.8) | 1 |
|  |  | Sal | Chicken |  |  |
| A1.6 | SXT^R^ | Ent | Pig | 1(0.4) | 1 |
| A1.7 | AZM^R^ | Cit | Pig | 1(0.4) | 1 |
| A1.8 | TE^R^ | Ent | Envmt | 1(0.4) | 1 |
| A1.9 | AX^R^ | Ent | Envmt | 2(0.8) | 1 |
|  |  | Ent | Envmt |  |  |
|  | Resistance to two antibiotic agents |  |  | 24 |  |
| A2.1 | SXT^R^ TE^R^ | Ent | Human | 1(0.4) | 2 |
| A2.2 | CFM^R^ ATM^R^ | Ent | Human | 1(0.4) | 1 |
| A2.3 | CAZ^R^ SXT^R^ | Ent | Human | 1(0.4) | 2 |
| A2.4 | NA^R^ ATM^R^ | Ent | Human | 1(0.4) | 2 |
| A2.5 | AX^R^ AM^R^ | Ent | Chicken | 4(1.8) | 1 |
|  |  | Ent | Human |  |  |
|  |  | Ent | Envmt |  |  |
|  |  | Ent | Envmt |  |  |
| A2.6 | FEP^R^ TE^R^ | Ent | Human | 1(0.4) | 2 |
| A2.7 | FEP^R^ SXT^R^ | Ent | Human | 1(0.4) | 2 |
| A2.8 | AMC^R^ AZM^R^ | Ent | Human | 1(0.4) | 2 |
| A2.9 | AMC^R^ AZM^R^ | Sal | Cattle | 1(0.4) | 1 |
| A2.10 | AM^R^ IPM^R^ | Cit | Human | 1(0.4) | 1 |
| A2.11 | FEP^R^ AM^R^ | Ent | Human | 1(0.4) | 1 |
| A2.12 | AX^R^ AMC^R^ | Sal | Pig | 4(1.8) | 1 |
|  |  | Sal | Pig |  |  |
|  |  | Ent | Envmt |  |  |
|  |  | Ent | Envmt |  |  |
| A2.13 | AMC^R^ ATM^R^ | Ent | Pig | 1(0.4) | 1 |
| A2.14 | AM^R^ AZM^R^ | Sal | Cattle | 1(0.4) | 2 |
| A2.15 | AM^R^ AMC^R^ | Sal | Human | 1(0.4) | 1 |
| A2.16 | CAZ^R^ AX^R^ | Ent | Envmt | 1(0.4) | 2 |
| A2.17 | AX^R^ AZM^R^ | Ent | Envmt | 1(0.4) | 2 |
| A2.18 | CFM^R^ AX^R^ | Ent | Envmt | 1(0.4) | 1 |
|  | Resistance to three antibiotic agents |  |  | 46 |  |
| A3.1 | **AX^R^ AM^R^ AMC^R^** | Ent | Human | **25**(11.1) | 1 |
|  |  | Cit | Human |  |  |
|  |  | Ent | Chicken |  |  |
|  |  | Ent | Chicken |  |  |
|  |  | Cit | Human |  |  |
|  |  | Ent | Human |  |  |
|  |  | Ent | Chicken |  |  |
|  |  | Ent | Chicken |  |  |
|  |  | Cit | Human |  |  |
|  |  | Ent | Human |  |  |
|  |  | Cit | Human |  |  |
|  |  | Cit | Human |  |  |
|  |  | Ent | Human |  |  |
|  |  | Sal | Chicken |  |  |
|  |  | Cit | Human |  |  |
|  |  | Ent | Human |  |  |
|  |  | Ent | Pig |  |  |
|  |  | Ent | Pig |  |  |
|  |  | Cit | Pig |  |  |
|  |  | Cit | Pig |  |  |
|  |  | Ent | Cattle |  |  |
|  |  | Sal | Chicken |  |  |
|  |  | Ent | Cattle |  |  |
|  |  | Ent | Envmt |  |  |
|  |  | Ent | Envmt |  |  |
| A3.2 | NA^R^ NOR^R^ ATM^R^ | Ent | Human | 1(0.4) | 2 |
| A3.3 | AX^R^ AM^R^ AZM^R^ | Ent | Human | 2(0.8) | 2 |
|  |  | Ent | Human |  |  |
| A3.4 | CAZ^R^ NA^R^ ATM^R^ | Sal | Human | 2(0.8) | 2 |
|  |  | Ent | Human |  |  |
| A3.5 | NA^R^ AM^R^ ATM^R^ | Ent | Human | 1(0.4) | 2 |
| A3.6 | CFM^R^ AX^R^ AMC^R^ | Ent | Human | 1(0.4) | 1 |
| A3.7 | NA^R^ ATM^R^ SXT^R^ | Ent | Human | 1(0.4) | 2 |
| A3.8 | FEP^R^ AX^R^ AMC^R^ | Cit | Human | 1(0.4) | 1 |
| **A3.9** | **AX^R^ AMC^R^ AZM^R^** | Ent | Human | **7**(3.1) | 2 |
|  |  | Ent | Human |  |  |
|  |  | Ent | Human |  |  |
|  |  | Ent | Human |  |  |
|  |  | Ent | Chicken |  |  |
|  |  | Ent | Pig |  |  |
|  |  | Sal | Envmt |  |  |
| A3.10 | CIP^R^ AX^R^ AMC^R^ | Ent | Human | 1(0.4) | 2 |
| A3.11 | CIP^R^ AM^R^ AMC^R^ | Cit | Chicken | 1(0.4) | 2 |
| A3.12 | AX^R^ AMC^R^ SXT^R^ | Ent | Human | 1(0.4) | 2 |
| A3.13 | AX^R^ AMC^R^ TE^R^ | Ent | Envmt | 1(0.4) | 2 |
| A3.14 | NOR^R^ AX^R^ AM^R^ | Ent | Envmt | 1(0.4) | 2 |
|  | Resistance to four antibiotic agents |  |  | 49 |  |
| A4.1 | CTX^R^ AX^R^ AM^R^ AMC^R^ | Ent | Human | 2(0.8) | 1 |
|  |  | Ent | Envmt |  |  |
| A4.2 | NA^R^ OFX^R^ ATM^R^ SXT^R^ | Cit | Human | 1(0.4) | 3 |
| A4.3 | CIP^R^ AX^R^ AM^R^ AMC^R^ | Ent | Human | 1(0.4) | 2 |
| A4.4 | CAZ^R^ AX^R^ AMC^R^ ATM^R^ | Ent | Human | 1(0.4) | 1 |
| **A4.5** | **CAZ^R^ AX^R^ AM^R^ AMC^R^** | Ent | Human | **12(5.3)** | **1** |
|  |  | Ent | Human |  |  |
|  |  | Cit | Human |  |  |
|  |  | Ent | Human |  |  |
|  |  | Ent | Cattle |  |  |
|  |  | Ent | Cattle |  |  |
|  |  | Ent | Cattle |  |  |
|  |  | Ent | Cattle |  |  |
|  |  | Ent | Cattle |  |  |
|  |  | Ent | Cattle |  |  |
|  |  | Ent | Cattle |  |  |
|  |  | Ent | Cattle |  |  |
| A4.6 | CAZ^R^ AM^R^ AMC^R^ SXT^R^ | Ent | Cattle | 1(0.4) | 2 |
| A4.7 | CAZ^R^ AX^R^ AM^R^ SXT^R^ | Ent | Cattle | 1(0.4) | 2 |
| **A4.8** | **AX^R^ AM^R^ AMC^R^ AZM^R^** | Ent | Human | **18(8.4)** | **2** |
|  |  | Sal | Human |  |  |
|  |  | Ent | Human |  |  |
|  |  | Ent | Human |  |  |
|  |  | Ent | Chicken |  |  |
|  |  | Ent | Human |  |  |
|  |  | Ent | Chicken |  |  |
|  |  | Ent | Chicken |  |  |
|  |  | Ent | Chicken |  |  |
|  |  | Cit | Pig |  |  |
|  |  | Cit | Pig |  |  |
|  |  | Sal | Pig |  |  |
|  |  | Cit | Pig |  |  |
|  |  | Ent | Chicken |  |  |
|  |  | Ent | Pig |  |  |
|  |  | Ent | Chicken |  |  |
|  |  | Ent | Envmt |  |  |
|  |  | Sal | Chicken |  |  |
| A4.9 | CFM^R^ AX^R^ AM^R^ AMC^R^ | Ent | Chicken | 1(0.4) | 1 |
| A4.10 | AM^R^ AMC^R^ SXT^R^ AZM^R^ | Ent | Human | 1(0.4) | 3 |
| A4.11 | CFM^R^ CAZ^R^ NA^R^ ATM^R^ | Ent | Cattle | 2(0.8) | 2 |
|  |  | Ent | Cattle |  |  |
| A4.12 | CAZ^R^ NA^R^ CIP^R^ ATM^R^ | Ent | Pig | 1(0.4) | 2 |
| A4.13 | AX^R^ AM^R^ AMC^R^ SXT^R^ | Ent | Envmt | 1(0.4) | 2 |
| A4.14 | CTX^R^ AX^R^ AM^R^ AMC^R^ | Sal | Human | 1(0.4) | 2 |
| A4.15 | AM^R^ AMC^R^ SXT^R^ TE^R^ | Ent | Human | 1(0.4) | 3 |
| A4.16 | CFM^R^ AX^R^ AMC^R^ ATM^R^ | Ent | Envmt | 1(0.4) | 1 |
| A4.17 | FEP^R^ AX^R^ AM^R^ AMC^R^ | Ent | Chicken | 2(0.8) | 1 |
|  |  | Ent | Cattle |  |  |
| A4.18 | AX^R^ AM^R^ AMC^R^ AK^R^ | Ent | Chicken | 1(0.4) | 2 |
|  | Resistance to five antibiotic agents |  |  | 24 |  |
| A5.2 | CAZ^R^ NA^R^ CIP^R^ AX^R^ AM^R^ | Cit | Human | 1(0.4) | 2 |
| A5.3 | CFM^R^ CAZ^R^ NA^R^ ATM^R^ AZM^R^ | Sal | Human | 2(0.8) | 3 |
|  |  | Ent | Cattle |  |  |
| A5.5 | CAZ^R^ AX^R^ AM^R^ AMC^R^ AZM^R^ | Ent | Cattle | 3(1.3) | 2 |
|  |  | Ent | Cattle |  |  |
|  |  | Ent | Human |  |  |
| A5.6 | CAZ^R^ NA^R^ CIP^R^ AMC^R^ SXT^R^ | Sal | Cattle | 1(0.4) | 3 |
| A5.7 | CAZ^R^ CTX^R^ AX^R^ AM^R^ AMC^R^ | Sal | Cattle | 1(0.4) | 1 |
| A5.8 | CAZ^R^ CTX^R^ NA^R^ AM^R^ AMC^R^ | Ent | Cattle | 1(0.4) | 2 |
| A5.9 | CAZ^R^ CTX^R^ AX^R^ AMC^R^ ATM^R^ | Ent | Human | 1(0.4) | 1 |
| A5.10 | CFM^R^ CAZ^R^ NA^R^ ATM^R^ SXT^R^ | Ent | Cattle | 2(0.8) | 3 |
|  |  | Sal | Human |  |  |
| A5.11 | AX^R^ AM^R^ AMC^R^ ATM^R^ SXT^R^ | Ent | Human | 2(0.8) | 2 |
|  |  | Ent | Human |  |  |
| A5.12 | FEP^R^ CFM^R^ AX^R^ AM^R^ AMC^R^ | Ent | Human | 1(0.4) | 1 |
| A5.13 | AX^R^ AM^R^ AMC^R^ AZM^R^ AK^R^ | Ent | Human | 2(0.8) | 3 |
|  |  | Sal | Human |  |  |
| A5.14 | AX^R^ AM^R^ AMC^R^ SXT^R^ AK^R^ | Ent | Chicken | 1(0.4) | 3 |
| A5.15 | AX^R^ AM^R^ AMC^R^ SXT^R^ AZM^R^ | Ent | Chicken | 1(0.4) | 3 |
| A5.16 | CFM^R^ CAZ^R^ CRO^R^ NA^R^ ATM^R^ | Ent | Human | 1(0.4) | 2 |
| A5.17 | CFM^R^ AX^R^ AM^R^ AMC^R^ AZM^R^ | Sal | Chicken | 1(0.4) | 2 |
| A5.18 | CRO^R^ NA^R^ OFX^R^ NOR^R^ CIP^R^ | Ent | Pig | 1(0.4) | 2 |
| A5.19 | CTX^R^ AX^R^ AM^R^ AMC^R^ AZM^R^ | Sal | Chicken | 1(0.4) | 2 |
| A5.20 | CAZ^R^ CRO^R^ AX^R^ AM^R^ AMC^R^ | Ent | Envmt | 1(0.4) | 1 |
|  | Resistance to six antibiotic agents |  |  | 12 |  |
| A6.1 | FEP^R^ CFM^R^ CAZ^R^ AX^R^ AM^R^ AMC^R^ | Ent | Human | 1(0.4) | 1 |
| A6.2 | CFM^R^ CAZ^R^ AX^R^ AM^R^ AMC^R^ SXT^R^ | Ent | Human | 1(0.4) | 2 |
| A6.3 | CFM^R^ CAZ^R^ CTX^R^ CRO^R^ AM^R^ AMC^R^ | Ent | Human | 1(0.4) | 1 |
| A6.4 | CFM^R^ NA^R^ AX^R^ AM^R^ AMC^R^ ATM^R^ | Ent | Chicken | 1(0.4) | 2 |
| A6.5 | CAZ^R^ CTX^R^ AX^R^ AM^R^ AMC^R^ ATM^R^ | Ent | Human | 2(0.8) | 1 |
|  |  | Sal | Human |  |  |
| A6.6 | CAZ^R^ AX^R^ AM^R^ AMC^R^ SXT^R^ AZM^R^ | Ent | Cattle | 1(0.4) | 3 |
| A6.7 | CAZ^R^ NA^R^ AX^R^ AM^R^ AMC^R^ SXT^R^ | Ent | Cattle | 1(0.4) | 3 |
| A6.8 | CRO^R^ AX^R^ AM^R^ AMC^R^ SXT^R^ TE^R^ | Ent | Chicken | 1(0.4) | 3 |
| A6.9 | NOR^R^ AX^R^ AM^R^ SXT^R^ C^R^ TE^R^ | Ent | Envmt | 1(0.4) | 5 |
| A6.10 | CTX^R^ CRO^R^ AX^R^ AM^R^ SXT^R^ C^R^ | Sal | Envmt | 1(0.4) | 3 |
| A6.11 | AX^R^ AM^R^ AMC^R^ SXT^R^ AZM^R^ AK^R^ | Ent | Envmt | 1(0.4) | 4 |
|  | Resistance to seven antibiotic agents |  |  | 10 |  |
| A7.1 | CFM^R^ CAZ^R^ NA^R^ AX^R^ AM^R^ AMC^R^ ATM^R^ | Ent | Human | 1(0.4) | 2 |
| A7.2 | CFM^R^ CAZ^R^ NA^R^ NOR^R^ CIP^R^ ATM^R^ SXT^R^ | Ent | Human | 1(0.4) | 3 |
| A7.3 | FEP^R^ CFM^R^ CAZ^R^ CTX^R^ CRO^R^ AM^R^ ATM^R^ | Ent | Human | 1(0.4) | 1 |
| A7.4 | CFM^R^ AX^R^ AM^R^ AMC^R^ ATM^R^ SXT^R^ TE^R^ | Sal | Chicken | 1(0.4) | 3 |
| A7.5 | CFM^R^ CAZ^R^ CTX^R^ CRO^R^ AM^R^ AMC^R^ ATM^R^ | Ent | Pig | 1(0.4) | 1 |
| A7.6 | NA^R^ CIP^R^ AX^R^ AM^R^ AMC^R^ SXT^R^ AZM^R^ | Ent | Chicken | 1(0.4) | 4 |
| A7.7 | CFM^R^ CAZ^R^ CTX^R^ AX^R^ AM^R^ AMC^R^ SXT^R^ | Ent | Envmt | 1(0.4) | 2 |
| A7.8 | FEP^R^ CFM^R^ CAZ^R^ CTX^R^ CRO^R^ AX^R^ AM^R^ | Ent | Envmt | 1(0.4) | 1 |
| A7.9 | CFM^R^ CRO^R^ NA^R^ AX^R^ AM^R^ ATM^R^ TE^R^ | Ent | Envmt | 1(0.4) | 3 |
| A7.10 | NA^R^ AX^R^ AM^R^ AMC^R^ SXT^R^ AZM^R^ TE^R^ | Ent | Envmt | 1(0.4) | 5 |
|  | Resistance to eight antibiotic agents |  |  | 12 |  |
| A8.1 | CFM^R^ CAZ^R^ CTX^R^ CRO^R^ NA^R^ AM^R^ AMC^R^ SXT^R^ | Ent | Human | 2(0.8) | 3 |
|  |  | Ent | Human |  |  |
| A8.2 | CAZ^R^ NA^R^ NOR^R^ AX^R^ AM^R^ AMC^R^ ATM^R^ AZM^R^ | Ent | Cattle | 1(0.4) | 3 |
| A8.3 | CFM^R^ CAZ^R^ CTX^R^ CRO^R^ NA^R^ ATM^R^ SXT^R^ AZM^R^ | Ent | Chicken | 1(0.4) | 4 |
| A8.4 | FEP^R^ CFM^R^ CAZ^R^ CTX^R^ CRO^R^ NA^R^ ATM^R^ TE^R^ | Cit | Chicken | 1(0.4) | 3 |
| A8.5 | CFM^R^ CAZ^R^ CRO^R^ NA^R^ AX^R^ AM^R^ ATM^R^ SXT^R^ | Ent | Human | 1(0.4) | 3 |
| A8.6 | CFM^R^ CAZ^R^ CRO^R^ NA^R^ AX^R^ AM^R^ AMC^R^ ATM^R^ | Ent | Chicken | 2(0.8) | 2 |
|  |  | Cit | Chicken |  |  |
| A8.7 | CFM^R^ NA^R^ OFX^R^ NOR^R^ CIP^R^ AX^R^ AM^R^ SXT^R^ | Ent | Pig | 1(0.4) | 3 |
| A8.8 | CFM^R^ CTX^R^ CRO^R^ AX^R^ AM^R^ AMC^R^ AZM^R^ AK^R^ | Ent | Pig | 1(0.4) | 3 |
| A8.9 | FEP^R^ CFM^R^ CAZ^R^ CTX^R^ CRO^R^ AX^R^ AMC^R^ SXT^R^ | Ent | Envmt | 1(0.4) | 2 |
| A8.10 | CFM^R^ CAZ^R^ CTX^R^ NA^R^ NOR^R^ CIP^R^ AX^R^ AMC^R^ | Ent | Envmt | 1(0.4) | 2 |
|  | Resistance to nine antibiotic agents |  |  | 12 |  |
| A9.1 | CFM^R^ CAZ^R^ CRO^R^ NA^R^ NOR^R^ AX^R^ ATM^R^ C^R^ AZM^R^ | Ent | Chicken | 1(0.4) | 4 |
| A9.2 | CFM^R^ CAZ^R^ CTX^R^ CRO^R^ AX^R^ AM^R^ AMC^R^ IPM^R^ AK^R^ | Ent | Human | 1(0.4) | 2 |
| A9.3 | CFM^R^ CAZ^R^ CTX^R^ CRO^R^ NA^R^ AX^R^ AM^R^ ATM^R^ SXT^R^ | Ent | Human | 1(0.4) | 3 |
| A9.4 | CFM^R^ CAZ^R^ NA^R^ NOR^R^ AX^R^ AM^R^ AMC^R^ ATM^R^ SXT^R^ | Ent | Human | 1(0.4) | 3 |
| A9.5 | CFM^R^ CAZ^R^ CRO^R^ NA^R^ AX^R^ AM^R^ AMC^R^ ATM^R^ AZM^R^ | Sal | Human | 1(0.4) | 3 |
| A9.6 | CFM^R^ CAZ^R^ CTX^R^ CRO^R^ AX^R^ AM^R^ AMC^R^ ATM^R^ SXT^R^ | Cit | Human | 3(1.3) | 2 |
|  |  | Ent | Chicken |  |  |
|  |  | Sal | Pig |  |  |
| A9.7 | FEP^R^ CFM^R^ CAZ^R^ CTX^R^ CRO^R^ AX^R^ AM^R^ AMC^R^ SXT^R^ | Ent | Human | 1(0.4) | 2 |
| A9.8 | CFM^R^ CTX^R^ CRO^R^ CIP^R^ AX^R^ AM^R^ AMC^R^ AZM^R^ TE^R^ | Ent | Cattle | 1(0.4) | 4 |
| A9.9 | NA^R^ OFX^R^ NOR^R^ CIP^R^ AX^R^ AM^R^ AMC^R^ SXT^R^ AZM^R^ | Ent | Envmt | 1(0.4) | 3 |
| A9.10 | CFM^R^ CAZ^R^ CTX^R^ NA^R^ NOR^R^ AX^R^ AM^R^ AMC^R^ SXT^R^ | Ent | Envmt | 1(0.4) | 3 |
|  | Resistance to ten antibiotic agents |  |  | 6 |  |
| A10.1 | FEP^R^ NA^R^ CIP^R^ AX^R^ AM^R^ AMC^R^ SXT^R^ C^R^ AZM^R^ TE^R^ | Ent | Human | 1(0.4) | 5 |
| A10.2 | CFM^R^ CAZ^R^ NA^R^ AX^R^ AM^R^ AMC^R^ ATM^R^ SXT^R^ AZM^R^ AK^R^ | Cit | Human | 1(0.4) | 5 |
| A10.3 | FEP^R^ CFM^R^ CAZ^R^ CTX^R^ CRO^R^ AX^R^ AM^R^ ATM^R^ SXT^R^ TE^R^ | Ent | Envmt | 1(0.4) | 3 |
| A10.4 | FEP^R^ CFM^R^ CAZ^R^ CTX^R^ CRO^R^ AX^R^ AM^R^ AMC^R^ ATM^R^ SXT^R^ | Ent | Envmt | 1(0.4) | 2 |
| A10.5 | CFM^R^ CAZ^R^ CTX^R^ NA^R^ NOR^R^ CIP^R^ AX^R^ AMC^R^ SXT^R^ C^R^ | Ent | Envmt | 1(0.4) | 4 |
| A10.6 | NA^R^ OFX^R^ NOR^R^ CIP^R^ AX^R^ AM^R^ AMC^R^ SXT^R^ C^R^ AZM^R^ | Ent | Envmt | 1(0.4) | 5 |
|  | Resistance to eleven antibiotic agents |  |  | 5 |  |
| A11.1 | FEP^R^ CFM^R^ CAZ^R^ CTX^R^ CRO^R^ NA^R^ AX^R^ AM^R^ AMC^R^ SXT^R^ AZM^R^ | Ent | Human | 1(0.4) | 4 |
| A11.2 | CFM^R^ CAZ^R^ CTX^R^ CRO^R^ NA^R^ AX^R^ AM^R^ AMC^R^ ATM^R^ SXT^R^ AZM^R^ | Sal | Human | 2(0.8) | 4 |
|  |  | Ent | Cattle |  |  |
| A11.3 | FEP^R^ CFM^R^ CAZ^R^ CTX^R^ CRO^R^ AX^R^ AM^R^ AMC^R^ SXT^R^ C^R^ TE^R^ | Cit | Pig | 1(0.4) | 4 |
| A11.4 | FEP^R^ CFM^R^ CAZ^R^ CTX^R^ CRO^R^ AX^R^ AM^R^ AMC^R^ ATM^R^ SXT^R^ TE^R^ | Ent | Envmt | 1(0.4) | 3 |
|  | Resistance to twelve antibiotic agents |  |  | 4 | 5 |
| A12.1 | CFM^R^ CAZ^R^ CTX^R^ CRO^R^ NA^R^ AX^R^ AM^R^ AMC^R^ ATM^R^ SXT^R^ AZM^R^ AK^R^ | Ent | Chicken | 1(0.4) | 5 |
| A12.2 | CFM^R^ CAZ^R^ CTX^R^ CRO^R^ NA^R^ OFX^R^ AX^R^ AM^R^ AMC^R^ ATM^R^ SXT^R^ AZM^R^ | Ent | Human | 2(0.8) | 4 |
|  |  | Ent | Human |  |  |
| A12.3 | FEP^R^ CFM^R^ CAZ^R^ CTX^R^ CRO^R^ AX^R^ AM^R^ AMC^R^ ATM^R^ SXT^R^ AZM^R^ TE^R^ | Ent | Envmt | 1(0.4) | 4 |
|  | Resistance to fourteen antibiotic agents |  |  | 1 |  |
| A14.1 | CFM^R^ CAZ^R^ CTX^R^ CRO^R^ NA^R^ OFX^R^ NOR^R^ CIP^R^ AX^R^ AM^R^ AMC^R^ C^R^ AZM^R^ AK^R^ | Ent | Envmt | 1(0.4) | 5 |
| **Total** | **131** |  |  | **214** |  |
|  | cefepime (FEP), cefixime (CFM), ceftazidime (CAZ), cefotaxime (CTX), ceftriaxone (CRO), nalidixic acid (NA), ofloxaxin (OFX), norfloxacin (NOR), ciprofloxacin (CIP), Amoxicillin (AX), Ampicillin (AM), Amoxicillin-Clavulanic acid(AMC), Imipenem (IPM), aztreonam (ATM), cotrimoxazole (SXT), chloramphenicol C, azithromycin (AZM), amikacin (AK), tetracycline (TE). *Enterobacter* (Ent), *Citrobacter* (Cit), *Salmonella* (Sal), Environment (Envmt) | | | | |
